# The Overexpression of TOB1 Induces Autophagy in Gastric Cancer Cells by Secreting Exosomes

**DOI:** 10.1155/2022/7925097

**Published:** 2022-04-12

**Authors:** Yanhong Wang, Ying Song, Lijie Zhou, Mengxi Wang, Dong Wang, Jing Bai, Songbin Fu, Jingcui Yu

**Affiliations:** ^1^Scientific Research Centre, The Second Affiliated Hospital of Harbin Medical University, Harbin 150081, China; ^2^Key Laboratory of Preservation of Human Genetic Resources and Disease Control in China (Harbin Medical University), Ministry of Education, Harbin 150081, China

## Abstract

We previously confirmed that transducer of ERBB2, 1 (TOB1) gene, can induce autophagy in gastric cancer cells. Studies have shown the biogenesis of exosomes overlaps with different autophagy processes, which helps to maintain the self-renewal and homeostasis of body cells. This study is aimed at verifying whether overexpressing TOB1 induces autophagy by secreting exosomes in gastric cancer cells and its underlying mechanisms. Differential ultracentrifugation was used to extracted the exosomes from the culture medium of gastric cancer cell line AGS-TOB1 ectopically overexpressing TOB1 (exo-AGS-TOB1, experimental group) and AGS-empty-vector cell line with low expression of endogenous TOB1 (exo-AGS-Vector, control group). Exosomal markers CD9 and TSG101 were determined in both the cell supernatants of exo-AGS-TOB1 and exo-AGS-Vector by Western blot. Under the transmission electron microscope (TEM), the exosomes were round and saucer-like vesicles with double-layer membrane structure, and the vesicles showed different translucency due to different contents. The peak size of exosomes detected by nanoparticle tracking analysis (NTA) was about 100 nm. When the exosomes of exo-AGS-TOB1 and exo-AGS-Vector were cocultured with TOB1 knockdown gastric cancer cell line HGC-27-TOB1-6E12 for 48 hours, the conversion of autophagy-related protein LC3-I to LC3-II in HGC-27-TOB1-6E12 gastric cancer cells cocultured with exo-AGS-TOB1 was significantly higher than that in the control group, and the ratio of LC3-II/LC3-I was statistically different (*P* < 0.05). More autophagosomes in HGC-27-TOB1-6E12 cells cocultured with exo-AGS-TOB1 for 48 hours were observed under TEM, while fewer autophagosomes were found in the control group. Lastly, miRNAs were differentially expressed by cell supernatant-exosomal whole transcriptome sequencing. Thus, our results provide new insights into TOB1-induced autophagy in gastric cancer.

## 1. Introduction

Gastric cancer is one of the common malignant tumors in the digestive system, with a high morbidity and mortality rate in China [1, 2]. The overall 5-year survival rate of gastric cancer patients is still less than 40% [3]. Therefore, understanding the molecular mechanism of gastric cancer occurrence and development is very important, which will help to promote the development of targeted drugs and the precise treatment of gastric cancer.

Exosomes are a class of circular extracellular vesicles with a lipid bilayer membrane, ranging in diameter from 30 to 150 nm [4]. Tumor cell-derived exosomes are capable of mediating tumor metastasis by transferring large amounts of their contents, including lipids, nucleic acids, and proteins, to adjacent and distant cells [5, 6]. Autophagy is a cellular self-digestion process, which maintains the dynamic balance of cells and tissues under the condition of lack of nutrition and stress, and participates in the regulation of tissue damage repair and fibrosis [7]. Activation of the autophagy mechanism leads to phagocytosis, digestion, and recycling of intracellular components [8, 9].

The interaction between exosomes and autophagy occurs in many different ways [10]. In the process of autophagy, autophagosomes can fuse with the lysosome [11]. When the intact autophagosomes fuse with the lysosome, the contents of the lysosomes are degraded by acid hydrolases, their components are recycled, and the autophagy pathway is completed [11]. In addition, autophagosomes can also fuse with intracellular multivesicular bodies (MVBs), which fuse with the cell membrane and release intraluminal vesicles as exosomes if the autophagic lysosomal pathway is impaired [12]. At the molecular level, autophagy-related proteins and protein complexes play a role in exosomal biogenesis [13]. At the organelle level, the exosomal and autophagy pathways overlap, and their contents have multiple fates, including extracellular release or lysosomal degradation [13]. When the autophagy degradation pathway is impaired, exosomal secretion is the most common alternative pathway for cells to relieve stress [14]. In addition, the autophagy process is also dependent on core autophagy receptors such as p62/SQSTM1, LC3, and GABARAPL2, which have been identified in the Vesiclepedia (http://www.microvesicles.org) database and vesicle experiment s [15]. Although the significance and role of exosomal signal transduction in tumors and the fact that autophagy occurs at multiple tumor stages have been extensively studied, the importance of the interaction between exosomes and autophagy in tumors is only just being recognized [16].

Transducer of ERBB2, 1 (TOB1) gene, is a member of the TOB/BTG antiproliferative protein family [17]. Previous study of our group first revealed that TOB1 is a gastric cancer-related tumor suppressor [18, 19]. Subsequently, our group has successively demonstrated that TOB1 is downregulated and phosphorylated in gastric cancer tissues [20], and TOB1 plays an antiproliferative role in the nucleus [21] and a series of studies [22–24]. Furthermore, the latest study of our group confirmed that TOB1 can induce autophagy in gastric cancer cells via decreasing the activation of AKT/mTOR signaling pathway [25], and the present study also proved for the first time that gastric cancer cells overexpressing TOB1 can induce autophagy in tumor cells by secreting exosomes.

## 2. Materials and Methods

### 2.1. Cell Line and Cell Culture

The human gastric adenocarcinoma cell lines AGS and HGC-27 were purchased from the American Type Culture Collection (ATCC, Manassas, VA, and USA) and the Shanghai Institute of Life Sciences, Chinese Academy of Sciences, respectively). Cell lines were authenticated by Beijing Microread Genetics Co., Ltd. (Beijing, China) using short tandem repeat analysis. AGS and HGC-27 were, respectively, cultured in F-12K medium (Gibco BRL, USA) and RPMI-1640 medium (Gibco BRL, USA) with 10% fetal bovine serum(FBS, PAA, Austria). In the previous study, we successfully constructed gastric cancer cell lines overexpressing ectopic TOB1 (AGS-TOB1) and knocking out TOB1 (HGC-27-TOB1-6E12). All of the above cell lines were cultured in a 37°C incubator with 5% CO_2_.

### 2.2. Western Blot Assay

Total protein was extracted from cells by using RIPA buffer containing protease and phosphatase inhibitors. The protein concentration was determined with a BCA Protein Assay Kit (Beyotime, Shanghai, China). Proteins were applied to SDS-PAGE (Beyotime Biotechnology, Nantong, Jiangsu, China) and then transferred to PVDF membranes (Immobilon™-PSQ Membranes, Sigma-Aldrich, Saint Louis, MO, USA). The membranes were blocked with 5% fat-free milk and hybridized with primary antibodies against TSG101 (Abcam, ab125011), CD9 (Abcam, ab92726), LC3B (Abcam, ab192890), and GAPDH (Proteintech, 60004-1-Ig) at 4°C, followed by secondary antibodies (anti-rabbit or anti-mouse antibodies, Rockland, Limerick, PA, USA). Immunoreactivity was detected with an Odyssey Infrared Imaging System (Li-COR, Lincoln, NE, USA) at wavelengths of 800 nm and 700 nm. ImageJ software was used to analyze the density value.

### 2.3. Exosome-Free Serum Preparation

Normal fetal bovine serum was aspirated into a sterile ultracentge tube and centrifuged at 120,000 rpm/min for 14 hours. The upper 60% volume serum in the centrifuge tube was sucked slowly, filtered with a 0.22 *μ*m filter membrane to obtain exosome-free serum, and stored at -20°C for later use.

### 2.4. Differential Ultracentrifugation to Extract Exosomes from Cell Supernatant

1.5 × 10^6^ cells were inoculated into a 15cm^2^ cell culture dish and added with ordinary medium. When the cell fusion rate reached 30% ~ 40%, the medium was discarded and replaced with 10% exosome-free serum medium after washing with PBS. When the cells were fused to more than 70%, the cell supernatant was collected, and the cell supernatant exosomes were extracted according to a differential ultracentrifugation method. In brief, at 4°C, 300 rpm/min for 10 minutes, 2,000 rpm/min for 10 minutes, 10,000 rpm/min for 30 minutes, and 120,000 rpm/min for 60 minutes, the exosomes precipitate at the bottom of the ultracentgation tube that was washed with PBS; the PBS suspension was centrifuged at 120,000 rpm/min for 60 minutes, and the precipitate was resuspended in PBS for later use.

### 2.5. Exosomes Cocultured with Gastric Cancer Cells

Gastric cancer cells were inoculated into a six-well plate at a density of 2.5 × 10^5^/well. Added 2 ml of normal medium and discarded the supernatant after culturing for 18 hours. After washing with PBS, exosome-free serum medium and extracted exosomes were added in a total volume of 2 ml per well. Cell pellets were collected after 48 hours for later use.

### 2.6. Nanoparticle Tracking Analysis

ZetaView PMX 110 nanoparticle tracking analyzer was used to perform nanoparticle tracking analysis on the extracted exosomes. The exosomes were appropriately diluted with 1× PBS and added to the sample wells of the instrument. The instrument randomly selected 11 different positions to determine the particle size and concentration of the samples in the wells. ZetaView measurement data was analyzed by ZetaView 8.04.02 software, and 110 nm polystyrene particles were used for Zetaview system calibration. The temperature was maintained at about 23°C ~ 30°C during the whole detection process.

### 2.7. Transmission Electron Microscopy

#### 2.7.1. Morphology and Structure of Exosomes

Dripped 15~ 20 *μ*l of exosomal suspension onto a copper mesh, let it stand for 5 ~ 10 minutes, and removed the excess liquid from the edge with filter paper. The phosphotungstic acid dye solution was dripped in for 1 ~ 2 minutes. The excess dye solution was absorbed by filter paper, and the images were observed and photographed under transmission electron microscope (Hitachi, Japan).

#### 2.7.2. Morphology and Structure of Autophagosomes

HGC-27-TOB1-6E12 cells cocultured with exo-AGS-TOB1 and exo-AGS-Vector for 48 hours were collected and centrifuged at 3,000 rpm/min for 15 minutes. The supernatant was discarded and fixed with 2.5% glutaraldehyde (Solarbio, Beijing, China) at 4°C for 2 hours, then washed with PBS, added 1% osmic acid, and fixed at 4°C for 2 hours. The mixture was washed with PBS, discarded the supernatant, and added acetone gradient for dehydration. The resin was saturated overnight at room temperature and polymerized at 70°C for 2 days. Leica microtome was used to slice, and the thickness was 70 nm. The slice was stained with uranyl acetate for 15 minutes, stained with lead citrate for 10 minutes, and observed under transmission electron microscope (Hitachi, Japan) after drying.

### 2.8. Cell Supernatant-Exosomal Whole Transcriptome Sequencing

Cell supernatants of exo-AGS-TOB1 and exo-AGS-Vector were collected. The supernatant after filtration was concentrated with a 100 KD ultrafiltration tube at 4°C and 3900 rpm/min for about 30 minutes. The concentrated solution was collected, quick-frozen in liquid nitrogen for 12 hours, and then stored at -80°C. About 400 ml of supernatant was concentrated to 40 ml. Novegene Bioinformatics Technology Co., Ltd. (Beijing, China) was commissioned to carry out the whole transcriptome sequencing and miRNA sequencing and analysis. The miRNA library construction process was as follows: highly sensitive Agilent 2100 pic600 was used to accurately detect the total amount and fragment distribution of RNA. After the samples were qualified, the library was constructed with Small RNA Sample Pre Kit, and the cDNA was reverse transcribed by adding adaptors at both ends of Small RNA. Subsequently, the target DNA fragments were separated by PCR amplification and PAGE gel electrophoresis, and the gel was cut and recovered to obtain a cDNA library. Qubit2.0 and Agilent 2100 were used for preliminary and accurate quantification, respectively. After the library was qualified, Illumina SE50 sequencing was performed.

### 2.9. Statistical Analysis

GraphaPad Prisim 8.0 software was used for statistical analysis of the data, and OriginPro 2017 software was used for mapping. The statistical data were calculated from three parallel experiments and expressed as mean ± standard deviation (*X* ± SD). Independent sample *t*-test was used to compare the difference among the groups, and *P* < 0.05 was considered as statistically significant.

## 3. Results

### 3.1. Exosomes Were Identified in the Culture Supernatants of Gastric Cancer Cells Overexpressing TOB1

**Figure 1:**
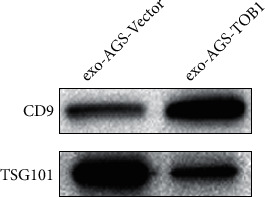
Western blot analysis of the expression of exosomal marker proteins CD9 and TSG101. The expression of CD9 and TSG101 could be detected in the exosomes extracted from the AGS culture medium of the control group and the experimental group with the ectopic overexpression of TOB1.

**Figure 2:**
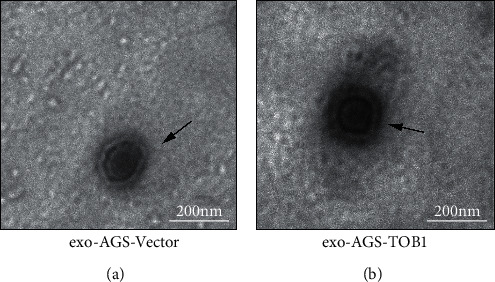
Morphological observation of exosomes by transmission electron microscopy in gastric cancer cells. (a) Morphological structure of exosomes of exo-AGS-Vector. (b) Morphological structure of exosomes of exo-AGS-TOB1 (scale bar = 200 nm).

**Figure 3:**
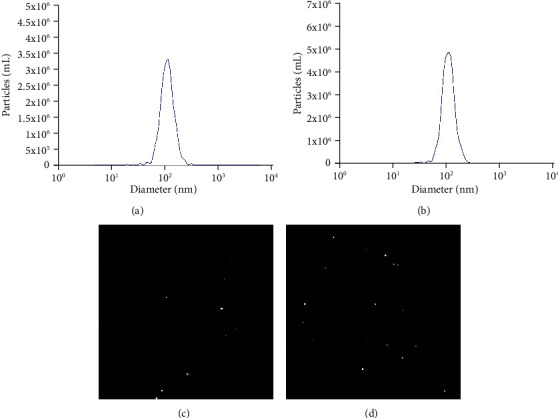
Nanoparticle tracking analysis of exosomes size distribution. (a) The peak diameter of exo-AGS-Vector purified exosomal particles was about 100 nm. (b) The peak diameter of exo-AGS-TOB1 purified exosomal particles was about 100 nm. (c) Brownian motion in exo-AGS-Vector purified exosomal particles. (d) Brownian motion in exo-AGS-TOB1 purified exosomal particles.

In order to explore the relationship between TOB1-induced autophagy and exosomes in gastric cancer cells, we selected gastric cancer cell lines overexpressing exogenous TOB1 (AGS-TOB1) and knocking out TOB1 (HGC-27-TOB1-6E12). According to the different sedimentation coefficients between exosomes and other organelles, ultracentrifugation was used to gradually remove cells and cell debris. Exosomes (exo-AGS-TOB1, experimental group) and exo-AGS-Vector (control group) were extracted from the culture medium of gastric cancer cell line AGS (donor cell) with ectopic overexpression of TOB1 and the culture medium of gastric cancer cell line AGS (donor cell) with low expression of endogenous TOB1. Proteins rich in exosomal membranes are often used as identification markers. CD9 (a member of the four transmembrane protein family) and tumor susceptibility gene 101 protein (TSG101) were selected as exosomal protein markers for Western blot analysis. As shown in Figure 1, CD9 and TSG101 proteins were detected in both exo-AGS-Vector and exo-AGS-TOB1 exosomes. Moreover, CD9 was enriched in exo-AGS-TOB1 exosomes while TSG101 was decreased in exo-AGS-TOB1 exosomes. Next, we used the transmission electron microscope (TEM) to observe the morphological structure of exo-AGS-Vector and exo-AGS-TOB1. As shown in Figure 2, both the exo-AGS-Vector-derived exosomes (Figure 2(a)) and the exo-AGS-TOB1-derived exosomes (Figure 2(b)) exhibited nearly round, saucer-like, bilayer membranous vesicles with diameters ranging from about 30 to 150 nm, and the transparency of the vesicles varied depending on their contents. Finally, the diameter distribution of exosomes was analyzed by nanoparticle tracking (NTA). The peak diameter of purified exosomes detected by NTA was about 100 nm (Figures 3(a) and 3(b)), which was in line with the diameter range of exosomes (30~ 150 nm). Tracking the Brownian motion trajectory of a single nanoparticle by NTA showed that both the purified exosomal particles exhibit irregular Brownian motion (Figures 3(c) and 3(d)).

### 3.2. Conversion of LC3-I Protein to LC3-II Protein Was Enhanced in Exosomes in the Culture Supernatants of Gastric Cancer Cells Overexpressing TOB1

**Figure 4:**
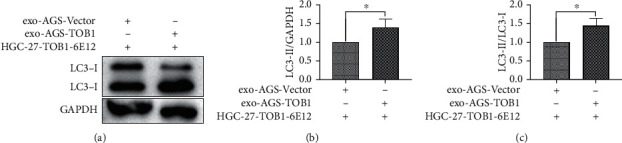
Coculture of exo-AGS-TOB1 with HGC-27-TOB1-6E12 cells for 48 hours increased the conversion of LC3-I protein to LC3-II protein in gastric cancer cells. (a) Western blot analysis of the LC3 protein expression in HGC-27-TOB1-6E12 cells. (b) The ratios of LC3-II/LC3-I protein by ImageJ analysis. (c) The ratios of LC3-II/GAPDH protein by ImageJ analysis.

Autophagy-related protein LC3 is involved in autophagosome biosynthesis in mammals and has been widely used as an autophagosome biomarker. When autophagosomes are formed, LC3-I will be transformed into LC3-II, and LC3-II can bind to the membrane of autophagosomes. Therefore, the level of autophagy can be evaluated by detecting the change of LC3-II/LC3-I level. In order to investigate whether exosomes secreted by gastric cancer cells overexpressing TOB1 can induce autophagy in gastric cancer cells, exosomes in the culture supernatants of gastric cancer cells AGS-TOB1 and AGS-Vector were cocultured with gastric cancer cells HGC-27-TOB1-6E12 (established by CRISPR/Cas9 technology in the early stage) for 48 hours, respectively. The expression of LC3-I and LC3-II in HGC-27-TOB1-6E12 cells was detected by Western blot. As shown in Figure 4, compared with the control group (exo-AGS-Vector + HGC-27-TOB1-6E12), LC3-I was significantly decreased, and LC3-II was significantly increased in the experimental group (exo-AGS-TOB1 + HGC-27-TOB1-6E12). The rate of conversion from LC3-I to LC3-II was significantly increased (^∗^*P* < 0.05, Figures 4(a) and 4(b)), and LC3-II/GAPDH was also statistically different (^∗^*P* < 0.05, Figure 4(c)). These results suggest that exosomes secreted by gastric cancer cells overexpressing TOB1 gene can promote tumor autophagy.

### 3.3. Exosome-Induced Autophagosomes Were Verified in the Culture Supernatants of Gastric Cancer Cells Overexpressing TOB1

**Figure 5:**
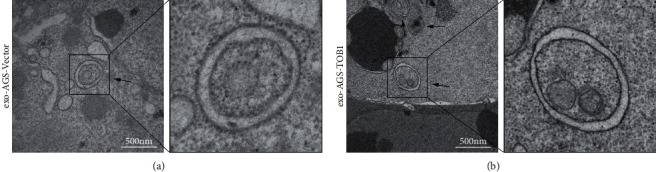
Transmission electron microscopic observation of autophagosomes in HGC-27-TOB1-6E12 gastric cancer cells cocultured with exo-AGS-TOB1. (a) Very few autophagosomes were observed in the control group of gastric cancer cells. (b) Many more autophagosomes were observed in the experimental group of gastric cancer cells (scale bar = 500 nm, receptor cell: HGC-27-TOB1-6E12).

Transmission electron microscope is the gold standard for the observation of autophagosomes. In order to further confirm that exosomes secreted by gastric cancer cells overexpressing TOB1 can induce autophagy in gastric cancer cells, we observed the ultrastructure of gastric cancer cells by TEM to find autophagosomes. As shown in Figure 5, under TEM, compared with the control group (Figure 5(a)), gastric cancer cells HGC-27-TOB1-6E12 cocultured with exo-AGS-TOB1 for 48 hours showed more typical autophagosomes with double membrane structure containing cytoplasmic components or organelles such as mitochondria or endoplasmic reticulum fragments to be digested and degraded (Figure 5(b)). These results further demonstrate that exosomes secreted by gastric cancer cells overexpressing TOB1 can induce autophagy in tumor cells.

### 3.4. Differentially Expressed miRNAs Were Enriched by Cell Supernatant-Exosomal Whole Transcriptome Sequencing

Our study hypothesized that the dysregulated RNA in the exosomes of gastric cancer cells overexpressing TOB1 might be transported to gastric cancer cells through the exosomal pathway to exert its biological role. miRNAs are small noncoding RNAs known to regulate gene expression by binding to the 3′ untranslated region of mRNA and partially complementing its target sequence. miRNAs inhibit or promote the translation of various oncogene or tumor suppressor mRNA, thereby affecting the biological function of cancer [26]. In order to obtain the differentially expressed miRNA molecules in the experimental group and control group of gastric cancer cells overexpressing TOB1, we performed whole transcriptome sequencing on exo-AGS-Vector and exo-AGS-TOB1 exosomes. Sequencing results showed that there were 9 differentially expressed miRNAs in exo-AGS-Vector and exo-AGS-TOB1. 1 miRNA (hsa-miR-12136) was upregulated in exo-AGS-TOB1 exosomes, and 8 miRNAs (has-miR-100-5p, has-miR-125b-1-3p, has-miR-146a-5p, has-miR-218-5p, has-miR-2682-5p, has-miR-3615, has-miR-452-5p, has-miR-548o-3p) were downregulated in exo-AGS-TOB1 exosomes (Table 1).

## 4. Discussion

Exosomes, as a powerful mediator of cell-to-cell communication, regulate the biological behavior of tumor and host cells locally within the tumor microenvironment and distally throughout the body by contacting recipient cells and transferring proteins, DNA, mRNA, and miRNA in their contents [27]. Many evidences indicate that the cellular response to maintain cell homeostasis is accomplished through the link between exosomes and autophagy [28]. Since both autophagy and exosomal formation and secretion are characterized by MVB formation, autophagy is closely related to exosomal release through the fusion of phagosomes with lysosomes or MVB [12]. Stress factors such as hypoxia have been shown to increase exosomal secretion and autophagy flux in breast cancer cells [29, 30]. Exosomes secreted by breast cancer cells are absorbed by human normal breast epithelial cell line (HMEC), and exosome-HMEC interactions can lead to the production of reactive oxygen species (ROS), which can induce autophagy and DNA damage response (DDR) in HMEC, and autophagic HMECs can release tumor-promoting factors [31]. Exosomes derived from CT26 (CT26Flag-CAGE) cultures of mouse colon cancer cells stably overexpressing the tumor/testis antigen CAGE could affect autophagy flux in mouse colon cancer cells via the CAGE-miR-140-5p-Wnt1 axis [32]. Exosomes derived from gefitinib-treated EGFR mutant lung cancer PC-9 cells could attenuate cisplatin response by increasing autophagy flux in the recipient cancer cells [33]. Thus, crosstalk between tumor-derived exosomes and autophagy affects the behavior of tumor cells and their interaction with the surrounding microenvironment.

As a member of the TOB/BTG antiproliferation protein family, TOB1 has the ability to inhibit cell proliferation and has an important impact on biological processes such as cell proliferation, cell cycle, growth, apoptosis, metabolism, and development by participating in the regulation of a variety of signaling pathways [34]. Many studies have shown that the abnormality of TOB1 gene is closely related to the occurrence and development of tumors [35]. TOB1 is the first gastric cancer-related tumor suppressor gene revealed by our research group [18–24], and our group also revealed in the previous study that overexpression of TOB1 in gastric cancer cells can induce autophagy in gastric cancer cells, and its molecular mechanism may be related to downregulation of AKT/mTOR signaling pathway [25]. Here, our study provides further evidence that exosomes secreted by gastric cancer cells overexpressing TOB1 can induce autophagy in gastric cancer cells.

First, we used ultracentgation [36] to separate and purify the exosomes secreted by gastric cancer cell line AGS overexpressing TOB1 gene and control gastric cancer cell line AGS. The data of protein markers detected by Western blot, exosomal morphology observed by TEM, and exosomal diameter detected by NTA proved that exosomes with high purity could be obtained by ultracentgation and used for subsequent coculture experiment analysis. To further explore the relationship between TOB1-induced autophagy and exosomes in gastric cancer, the exosomes secreted by gastric cancer cells overexpressing TOB1 (exo-AGS-TOB1) and control gastric cancer cells (exo-AGS-Vector) were cocultured with TOB1-knockout gastric cancer cell line HGC-27-TOB1-6E12 for 48 hours. Western blot was used to analyze the expression of autophagy-related protein LC3 in HGC-27-TOB1-6E12 cells, and TEM was used to observe the formation and number of autophagosomes to determine the occurrence of autophagy in gastric cancer cells. LC3 has two forms in cells, LC3-I and LC3-II [37], which is involved in autophagosome biosynthesis in mammals, and is widely used as a biomarker of autophagy. When autophagy occurs, LC3-I is modified and processed to covalently bind to phosphatidylethanolamine (PE) to form LC3-II, which is located on the autophagosomal membrane. The expression of LC3-II is closely related to the number of autophagosomes; so, comparing the expression of LC3-II can accurately reflect the level of autophagy [38]. In addition, the ratio of LC3-II/LC3-I is a marker reflecting the occurrence of autophagy, and the higher the ratio, the higher the autophagy flux. In this study, we detected a significant increase in the level of LC3-II protein expression and a significant decrease in the level of the LC3-I protein expression in HGC-27-TOB1-6E12 cells cocultured with exo-AGS-TOB1 for 48 hours and a significant increase in the transition from LC3-I to LC3-II. The ratios of LC3-II/LC3-I and LC3-II/GAPDH were significantly higher than those in the control group. These data provide evidence that exosomes secreted by gastric cancer cells overexpressing TOB1 increase autophagy of LC3-II accumulation.

The hallmark structure of autophagy is the formation of autophagosomes; so, the observation of autophagosome under TEM is the “gold standard” to detect autophagy [25]. Autophagosomes have a bilayer or multilayer membrane structure and contain cytoplasmic components or organelles (such as mitochondria or endoplasmic reticulum fragments) to be digested and degraded [39–41]. In our study, typical autophagosomes were detected in HDC-27-TOB1-6E12 cells cocultured with exo-AGS-TOB1 for 48 hours, while few or no autophagosomes were detected in control cells. These results further provided evidence that exosomes secreted by gastric cancer cells overexpressing TOB1 can induce autophagosome production in gastric cancer cells.

The above studies have confirmed that the overexpression of TOB1 in gastric cancer cells can induce autophagy by secreting exosomes, but the specific mechanism remains to be further verified. The PI3K/AKT signaling pathway is the primary pathway for cell survival and is highly activated in a variety of tumor tissues [42]. mTOR is a key negative regulator of autophagy, a serine/threonine protein kinase, which can regulate cell growth, cell proliferation and protein synthesis [43]. Downregulation of PI3K/AKT leads to mTOR inactivation and induces autophagy in cancer cells. Therefore, the PI3K/AKT/mTOR signaling pathway is a classic way to regulate autophagy [44, 45] and plays an important role in antitumor by regulating autophagy activity. Peng et al. found that blocking PI3K/AKT/mTOR signaling pathway can enhance apoptosis and autophagy and induce G1 cell cycle arrest, thereby inhibiting the proliferation of colon cancer cell HCT116 [46]. The anticancer drug sotrexoflavone induces autophagy in non-small-cell lung cancer A549 by blocking the PI3K/AKT/mTOR signaling pathway, accelerating A549 cell death [47]. This pathway plays an important role in tumors and has now become a research hotspot of tumor molecular marker-targeted therapy [48, 49]. Regarding the relationship between TOB1 and PI3K/AKT/mTOR signaling, Zhang et al. found that knocking out TOB1 gene in estrogen-dependent breast cancer cell MCF-7 can activate a downstream target of ERBB2 signaling, and ultimately, estrogen-independent cells are more sensitive to AKT and mTOR inhibitors [50]. The latest study of our group confirmed that TOB1 can induce autophagy in gastric cancer cells via decreasing the activation of AKT/mTOR signaling pathway [25].

Our group subsequently identified 9 differentially expressed miRNAs (1 miRNA upregulated and 8 miRNAs downregulated) in the exosomal contents of the culture supernatant of gastric cancer cells overexpressing TOB1. Among them, miR-218-5p is a tumor suppressor miRNA, which can inhibit the progression of retinoblastoma by targeting NACC1 to inhibit AKT/mTOR signaling pathway [51]. MiR-100-5p is significantly low expressed and is a potential biomarker of prostate cancer [52]. Also, as a tumor suppressor miRNA, miR-100-5p can inhibit the proliferation, migration, and invasion of prostate cancer cells by downregulating the expression of mTOR [53]. In gastric cancer cells, lncRNA HAGLROS competitively binds to miR-100-5p to increase the mTOR expression by antagonizing miR-100-5p-mediated mTOR inhibition, thereby inhibiting autophagy and promoting malignant progression of gastric cancer cells [54]. In addition, exosomes derived from cisplatin-resistant lung cancer cells have been found to use mTOR as a potential target to confer resistance to cisplatin on recipient cells in an exosomal miR-100-5p-dependent manner [55]. MiR-2682-5p is a miRNA that is downregulated in the exosomes of exo-AGS-TOB1. As a tumor suppressor, exosome-derived miR-2682-5p can inhibit the viability and migration of non-small-cell lung cancer cells and promote apoptosis through the HDAC1/ADH1A axis [56]. At the same time, SNHG3/miR-2682-5p/HOXB8 axis, ELK1/lncRNA-SNHG7/miR-2682-5p feedback loop, and LINC01006/miR- 2682-5p/HOXB8 axis can promote the proliferation and migration of oral squamous cell carcinoma [57], bladder cancer [58], and pancreatic cancer [59], respectively. miR-452-5p is a miRNA with dual functions of tumor suppressor and oncomiR. Lin et al. found that miR-452-5p is upregulated in colorectal cancer and contributes to the progression of colorectal cancer by activating the miR-452-5p-PKN2/DUSP6-c-Jun positive feedback loop [60]. On the contrary, Yan et al. found that miR-452-5p was significantly downregulated in colorectal cancer tissues and promoted the development, invasion, and metastasis of colorectal cancer [61]. In recent years, miRNAs have been widely recognized in cancer treatment and diagnosis [62]. Study has shown that the miR-452-5p expression is downregulated after sunitinib treatment and inhibits the migration and invasion of renal cancer cells by regulating the SMAD4/SMAD7 signaling pathway [63]. Exosome-derived miR-452-5p can be used for noninvasive diagnosis and treatment monitoring of esophageal adenocarcinoma [64]. miR-125b-1-3p plays an antitumor role in lung cancer [65], while exosome-derived miR-125b-1-3p is an active component that mediates skeletal muscle atrophy during cancer cachexia [66]. In addition, miR-125b-1-3p [67], miR-146a-5p [68], miR-3615 [69], and miR-12136 [70] can also be used as noninvasive biomarkers of cancer. miR-548o-3p has conducted predictive studies in type 1 diabetes [71] and cardiovascular disease [72] but has not been reported in cancer research. Therefore, our study further speculated that the overexpression of TOB1 in gastric cancer cells affects the expression of characteristic miRNA molecules represented by miR-218-5p, miR-100-5p, miR-2682-5p, miR-452-5p, miR-125b-1-3p, miR-146a-5p, miR-3615, miR-12136, and miR-548o-3p, which enters gastric cancer cells through the exosomal pathway and exerts biological functions including autophagy.

However, the major limitation of the present study is that the specific molecular mechanism of the biological function of the above nine differentially expressed miRNAs is not clear. At present, previous studies of our group have revealed that the overexpression of TOB1 can induce autophagy in gastric cancer cells, and its mechanism may be related to the down-regulation of Akt/mTOR signaling pathway. Therefore, future research will be focused on the differential expression of PI3K/Akt/mTOR pathway-related proteins in gastric cancer cells and their interaction with exosome-derived differentially expressed miRNAs to explore the potential molecular mechanism of autophagy induced by exosomes secreted by gastric cancer cells overexpressing TOB1.

## 5. Conclusion

In conclusion, the current results strongly confirm that gastric cancer cells overexpressing TOB1 gene induce autophagy by secreting exosomes. In addition, we speculated that differentially expressed miRNAs in the exosomal content were taken up by the receptor cells, which might affect the activity of PI3K/Akt/mTOR pathway, and then induce autophagy in gastric cancer cells with TOB1 gene knockout. More evidence is needed to study the underlying mechanism of its occurrence. This study is also expected that the differentially expressed miRNAs, represented by miR-218-5p, miR-100-5p, miR-2682-5p, miR-452-5p, miR-125b-1-3p, miR-146a-5p, miR-3615, miR-12136, and miR-548o-3p, may be used as diagnostic or prognostic biomarkers for cancer patients. In addition, the functional miRNAs in the exosomes secreted by gastric cancer cells overexpressing TOB1 need to be further explored to provide more candidate molecules for tumor diagnosis and treatment.

## Figures and Tables

**Table 1:** Differentially expressed miRNAs in exo-AGS-Vector and exo-AGS-TOB1.

	Log2 fold change	*P* val.
hsa-miR-12136	3.222486813	0.038687116
has-miR-100-5p	-2.095924928	0.022333591
has-miR-125b-1-3p	-4.882062404	0.016786292
has-miR-146a-5p	-3.027796596	0.022089101
has-miR-218-5p	-2.907375196	0.019030972
has-miR-2682-5p	-6.827730485	0.004934552
has-miR-3615	-2.866919075	0.019477919
has-miR-452-5p	-6.19339564	0.015831514
has-miR-548o-3p	-1.231721903	0.036541339

## Data Availability

All data used to support the findings of this study are available from the corresponding author upon request.
